# Efficacy of Adaptol^®^ 500 mg Tablets in Patients with Anxiety and Somatic Symptoms of Anxiety Disorder: A Noninterventional Study

**DOI:** 10.3390/jcm14227972

**Published:** 2025-11-10

**Authors:** Maris Taube, Guna Dansone, Yulia Troshina

**Affiliations:** 1 Department of Psychosomatic Medicine and Psychotherapy, Riga Stradiņš University, 1083 Riga, Latvia; 2 National Center of Mental Health, 1005 Riga, Latvia; 3 Medical and Clinical Research Department, JSC Olpha, 2114 Olaine, Latvia

**Keywords:** Adaptol^®^, temgicoluril, anxiety, somatic symptoms, efficacy, treatment

## Abstract

**Background**: Anxiety disorders, including panic disorder, agoraphobia, specific phobias, and generalized anxiety disorder, are among the most frequent psychiatric conditions in primary care. They often present with somatic symptoms such as dyspnea, palpitations, chest or gastrointestinal discomfort, sweating, or flushing. Adaptol^®^ is a non-benzodiazepine anxiolytic with nootropic properties that modulates the limbic-reticular system, hypothalamic emotional centers, and multiple neurotransmitter systems. This study aimed to assess the association between Adaptol^®^ use and changes in anxiety symptoms, including somatic manifestations, in routine practice. **Methods**: A noninterventional observational study was conducted in 100 adults diagnosed with anxiety disorders in primary care. All received Adaptol^®^ 500 mg as prescribed. Patients had to have mild-to-moderate anxiety (5–14 points according GAD-7) to be enrolled. Exclusion criteria ruled out individuals with concomitant psychiatric or severe somatic conditions and those with use of other medications or any interventions that could affect the symptoms. Anxiety severity and somatic symptom burden were assessed at baseline and after treatment. **Results**: Adaptol^®^ treatment was associated with reduction in anxiety and somatic complaints. Improvements were reported in palpitations, chest discomfort, gastrointestinal disturbances, and autonomic symptoms. Greater benefit was observed in male patients, though without significance testing, and in those with severe baseline anxiety, as demonstrated by correlation between GAD-7 scores at baseline and changes after the treatment (r = 0.5). No unexpected adverse events occurred. **Conclusions**: In this real-world study, Adaptol^®^ showed anxiolytic efficacy and good tolerability, improving both psychological and somatic manifestations of anxiety disorders. These findings support its use in primary care, especially in severe cases of anxiety. Controlled trials are needed to support these results.

## 1. Introduction

### Background

Anxiety disorders are a common issue seen in the primary healthcare setting. They include panic disorders, specific phobias, and generalized anxiety disorders. The diagnostic criteria for anxiety disorders include symptoms from different organ systems (somatic symptoms) based on the *Diagnostic and Statistical Manual of Mental Disorders, fifth edition*. In addition to somatic manifestations, anxiety is associated with psychological and behavioural correlates, such as cognitive distortions, maladaptive coping patterns, and avoidance behaviours, highlighting its multifactorial nature. Somatic symptoms of anxiety by organ systems include cardiopulmonary (feeling short of breath, palpitations, and chest discomfort), gastrointestinal (nausea or gastrointestinal discomfort), and the skin (sweating, chills, or feeling flushed) manifestations [1,2,3].

The primary therapeutic indications of Adaptol^®^ (with the active substance temgicoluril; ATC code N06BX21) in adults include neurosis and neurotic disorders, e.g., anxiety, unrest, fear, emotional lability, and also cardialgia that is not associated with coronary heart disease [4]. Temgicoluril has anxiolytic and nootropic properties. It affects activity of the limbic-reticular system, as well as four basic neurotransmitter systems: γ aminobutyric acid, the choline, serotonin, and adrenergic systems; however, it has no peripheral adrenolytic activity. Temgicoluril demonstrates moderate anxiolytic activity, and eliminates or reduces feelings of unrest, anxiety, fear, inner emotional tension, and irritability. The tranquilizing effect of temgicoluril is not accompanied by myorelaxation, movement coordination disorder, or mental and physical activity suppression. Thus, it can be used without interfering with work or study. Although temgicoluril has no direct sedative effects, it increases hypnotic activity and normalizes disturbed sleep patterns. Temgicoluril also reduces the unfavorable side effects induced by neuroleptics and tranquilizers of the benzodiazepine group (e.g., depressed mood, an excessive sedative effect, and muscle weakness). In addition, temgicoluril does not cause an elevated mood or euphoria, and no tolerance, drug dependence, or withdrawal syndrome has been observed during its administration. Furthermore, clinical studies have demonstrated that temgicoluril increases logical and associative thinking and improves attention and work capacity without stimulating symptoms such as delirium or abnormal emotional activity [5]. Recent research has also reported beneficial effects of Adaptol^®^ in complex clinical settings, such as in elderly patients with combined arterial hypertension and postinfectious syndrome, where the drug contributed to reducing anxiety and improving overall treatment tolerance. These findings support the clinical relevance of evaluating Adaptol efficacy in anxiety management, including potential differences by sex and age [6]. While temgicoluril demonstrates anxiolytic activity and favourable tolerability, it is important to note that current clinical guidelines recommend multimodal approaches to anxiety management, combining pharmacotherapy with psychological interventions such as cognitive–behavioural therapy. In routine clinical practice, Adaptol^®^ may be used as part of an integrated treatment plan, rather than as a standalone therapy.

Therefore, this study aimed to evaluate the efficacy of Adaptol^®^ for managing anxiety in adult outpatients. There was a focus on somatic symptoms and the correlation between the anxiety severity and treatment outcomes, as well as age and sex differences in response to the medication therapy. Psychological variables, such as coping patterns or cognitive distortions, were not assessed in this study, which should be considered a limitation when interpreting the findings.

## 2. Materials and Methods

### 2.1. Subject

A non-interventional observational study was conducted across several general practice offices in Latvia.

### 2.2. Materials

This was an observational study that collected data on adult outpatients with anxiety classified under ICD-10 diagnostic codes F40–F48, which encompass neurotic, stress-related, and somatoform disorders [7], and who were prescribed 500 mg Adaptol^®^ tablets in accordance with typical clinical practice and the approved product specification. The study was designed in collaboration with a certified psychiatrist and conducted in several general practice offices, who showed willingness to participate, throughout Latvia. The study was reviewed and approved by the Medical and Biomedical Research Ethics Committee of the Riga Eastern Clinical University Hospital Support Fund. All participants provided written consent for the use of their medical data. Detailed information about the nature of the study, research methods, protocol conditions, patients’ rights, and personal data protection was provided in the informed consent document and discussed during the consent process.

Adult outpatients (18–70 years old) prescribed 500 mg Adaptol^®^ tablets two times per day for at least three weeks for anxiety in accordance with approved prescription information were considered for this study. Patients had to have mild-to-moderate anxiety (5–14 points according to a Generalized Anxiety Disorder 7-item scale (GAD-7) [5]) and at least two somatic symptoms of anxiety disorder (shortness of breath, palpitations, chest discomfort, nausea, gastrointestinal discomfort, sweating, chills, or feeling flushed) that could not be explained by known physical or another psychiatric disorder. Exclusion criteria ruled out individuals with concomitant psychiatric or severe somatic conditions and those with the use of medications or any interventions that could affect the symptoms. Prior to enrollment, patients were examined by a general practitioner, and their eligibility was confirmed based on their current clinical presentation, medical history and GAD-7 score. Patients completed the GAD-7 questionnaire at the start of their prescription and after the treatment course.

The GAD-7 is a seven-item self-assessment scale used to measure the severity of generalized anxiety disorder. Each item asks the individuals to rate the severity of their symptoms over the past two weeks. Response options include “not at all”, “several days”, “more than half the days” and “nearly every day” (scoring 0, 1, 2, and 3 points, respectively). GAD-7 total scores range from 0 to 21. Scores 5, 10, and 15 represent the cut-points for mild, moderate, and severe anxiety, respectively. The minimal clinically significant difference is considered to be 4 points [8,9].

In addition, a doctor provided clinical judgement whether a patient’s somatic symptoms had improved after the treatment course using one of the following ratings: “very much improved”, “much improved”, or “not improved”. Adverse events had to be reported to the country’s State Agency of Medicines, as per national requirements.

### 2.3. Methods

#### 2.3.1. Data Collection

Individual, paper-based, case report forms were used to collect the following data: a unique participant number; informed consent date; age; sex; compliance with protocol criteria; specific somatic symptoms; GAD-7date administered, point-by-point values, and total score before and after the treatment course; the number and days of Adaptol^®^ 500 mg tablets taken by each patient; other concomitant treatments used; improvement of somatic symptoms; adverse events.

#### 2.3.2. Statistical Methods

A formal sample-size calculation was not performed because this study was exploratory and intended to generate hypotheses and estimate effect sizes for future research. Up to 100 subjects were planned to be involved, respecting a real-time patient flow and project time frame for the final number. Changes in GAD-7 scores and somatic symptoms after the three-week treatment course were presented as absolute and relative frequencies. The subgroup data are provided only descriptively. The change in GAD-7 before and after the treatment course was evaluated at the 5% significance level using a paired *t*-test. Data analysis was performed using IBM^®^ SPSS Statistics ver. 29 (IBM Corp, Armonk, New York, NY, USA).

## 3. Results

### 3.1. Demographic and Clinical Characteristics of the Study Participants

**Figure 1:**
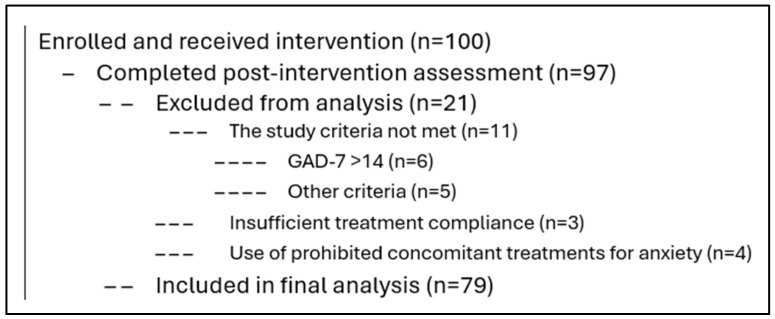
Participant flow.

In total, 100 patients participated in the study between June 2023 and May 2024. Patients who had taken 500 mg Adaptol^®^ tablets twice a day for three weeks (21 ± 3 days, a total of at least 36 tablets) and had pre- and post-treatment GAD-7 data available were included in the statistical analysis. Twenty-one patients were excluded from the primary analysis. For six patients, the study criteria were not met, with GAD-7 scores >14 points. For five patients, other criteria were not met. Three patients quit the study or were lost to follow-up, another three demonstrated insufficient compliance with the study protocol, and four patients used other, concomitant treatments for anxiety that were not acceptable according to the study protocol. The six patients who had severe anxiety were reviewed separately (Figure 1).

### 3.2. Primary Analysis

**Figure 2:**
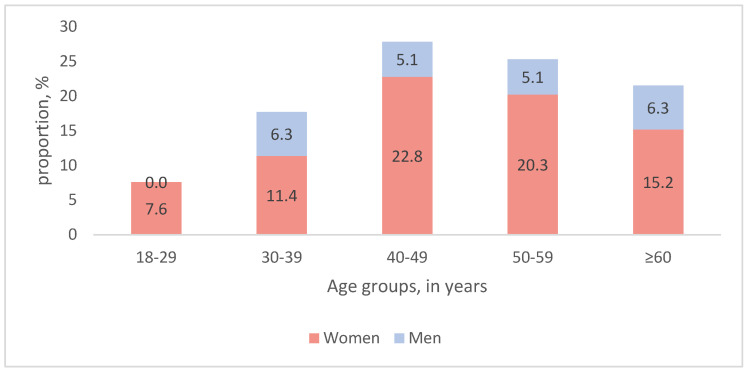
Age and sex of the study population (n = 79).

Seventy-nine patients (61 women and 18 men) were included in the primary analysis. The mean age was 48 years (±13 years, 18–70) (Figure 2). The mean GAD-7 score before the treatment was 10.9 points (±2.7, 5–14). According to the GAD-7 results, 25% (20/79) of patients had mild (5–9 points) and 75% (59/79) had moderate anxiety (10–14 points).

Ninety-five percent of the participants reported somatic symptoms related to the cardiovascular system (shortness of breath, palpitations, and chest discomfort). Somatic symptoms related to the gastrointestinal system (nausea and gastrointestinal discomfort) and the skin (sweating, chills, feeling flushed) were reported by 40.5% and 63.3% of patients, respectively. Approximately half of the patients (50.6%) had symptoms involving two organ systems, while a quarter of participants had symptoms in one (25.3%) or all three systems (24.1%). The most frequent combination was cardiovascular and skin symptoms (34.2%).

Seventy-seven patients took a total of 40 Adaptol^®^ 500 mg tablets over three weeks. Two patients used 38 tablets. The mean time between baseline and post-treatment assessments was 22 days (±4), with the most common time frame between 20 and 28 days (76 patients). However, for two patients it was 19 and 55 days.

### 3.3. Effects of Adaptol^®^ Treatment

After treatment, the mean GAD-7 score decreased to 6.4 points (±3.6, range 0–17). This was significantly different from the initial mean value of 10.9 points (*p* < 0.05; two-tailed, paired *t*-test). The standardized effect size (Cohen’s d) was 1.34 (95% CI 1.04–1.64), which supports the intervention’s strong impact on the observed outcome. The mean change was 4.5 points (±3.4, min–max −13 and −5), which is a clinically significant change.

**Figure 3:**
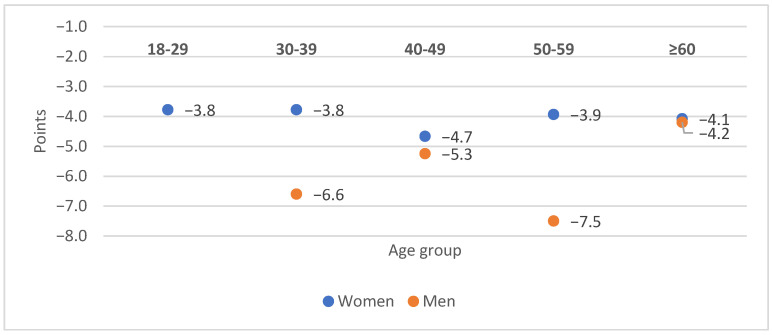
The mean change in GAD-7 scores depending on patient age and sex.

In all age groups, anxiety decreased more in men than women, with the highest response in 50–59-year-old men (mean GAD-7 of −7.5 points), followed by 30–39-year-olds (−5.3 points). The highest response in women was observed in the 40–49-year-olds (mean GAD-7 of −5.3 points), followed by the over-60 group (−4.2 points) (Figure 3).

**Figure 4:**
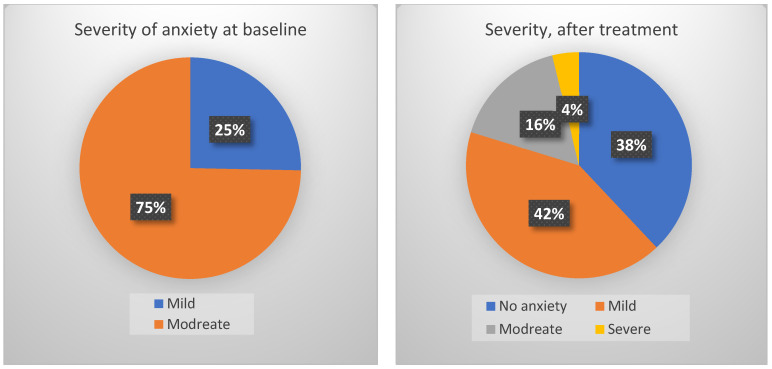
Visual representation of anxiety severity before and after the three-week treatment course according to GAD-7 scores.

A minimal clinically significant improvement (4 points on the GAD-7) was observed in 67% (53/79) of the patients. The most frequent change was from moderate to mild anxiety (47%) (Table 1). For four patients (5%), their anxiety worsened, and for three patients (4%), the GAD-7 score remained the same (Figure 4).

**Figure 5:**
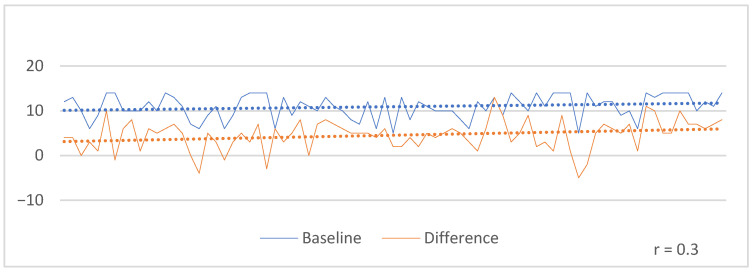
Correlation between GAD-7 baseline scores and after the treatment course.

There was no correlation between changes in the GAD-7 and patient age (Pearson’s correlation coefficient, r = 0.02). However, there was a weak positive correlation between GAD-7 scores at baseline and changes after treatment (r = 0.3) (Figure 5).

**Figure 6:**
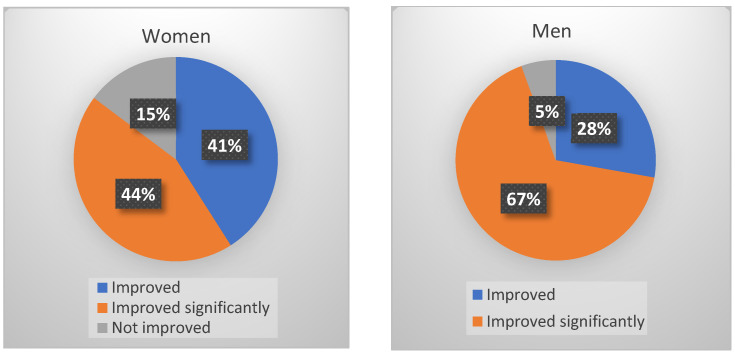
Somatic symptoms after the three-week treatment course.

A decrease in somatic symptoms was reported for 87% of the study participants. The decrease was more prominent in men than women, with significant improvement occurring in 67% of men vs. 44% of women (Figure 6).

There was no correlation between the baseline GAD-7 scores and the number of involved systems or the improvement of somatic symptoms. However, changes in somatic symptoms were correlated with changes in the GAD-7 score. The mean change in GAD-7 scores for participants with improved somatic symptoms, those whose symptoms improved significantly, and those who did not experience improved symptoms were −3.3, −7, and 1.4 points, respectively.

### 3.4. Patients with Severe Anxiety

Six patients with severe anxiety at baseline (a GAD-7 score of 18–19) were found to be ineligible after study inclusion and having received a full treatment course. These patients were reviewed separately. Significant improvement was observed in all six individuals (Table 2).

When adding the data from these patients to the correlation analyses, the positive correlation between GAD-7 scores at baseline and changes after the treatment course went from weak to moderate (r = 0.5).

### 3.5. Concomitant Treatments

Approximately one-third of the patients received concomitant medications, mostly for cardiovascular, gastrointestinal, and endocrine systems. Some participants used over-the-counter medicines or vitamins. Nonpharmacologic interventions like exercise and therapeutic massage were reported for three patients with severe anxiety who were reviewed separately from the per protocol analysis set. Patients who received medicines not permitted by the study protocol (e.g., benzodiazepines and gabapentin) were excluded from the statistical analysis.

## 4. Discussion

Our results confirmed that Adaptol^®^ was associated with pronounced improvements in anxiety under the specified dose regimen. Only 9% of patients did not experience any improvement in anxiety, while 67% with mild-to-moderate anxiety and 100% of those with severe anxiety demonstrated clinically significant improvement. Somatic symptoms improved in 87% of patients with mild-to-moderate symptoms and in 100% of those with severe symptoms. Improvements in somatic symptoms were concomitant with a decrease in GAD-7 scores. In general, these observations support the available data on Adaptol^®^. These findings suggest that the observed benefits of Adaptol^®^ may reflect interactions between biological effects, individual psychological coping mechanisms, and social-contextual factors, consistent with a biopsychosocial framework. While the concomitant improvements in somatic and psychological symptoms suggest a potential benefit of Adaptol^®^, they may also reflect general symptom relief rather than a specific drug effect. Such improvements could also result from cross-level regulatory mechanisms and other conceptual confounders, including patient expectancies and agency-related appraisals (e.g., perceived control, coping resources), as well as environmental factors (e.g., social support, workload, access to care), all of which may modulate symptom trajectories independently of pharmacology, in line with a biopsychosocial framework [10]. Previous studies have demonstrated similar anxiolytic and somatic symptom–reducing effects of Adaptol^®^ in adult populations, including elderly patients and those with comorbid cardiovascular conditions [5,6]. These findings are consistent with our results, supporting the observed tolerability and potential benefit of temgicoluril across different patient groups. Although patients with severe anxiety (a GAD-7 score >14) were not part of the primary analysis, Adaptol^®^ use was associated with marked reductions in anxiety symptoms in these patients. Moreover, a correlation analysis of pooled data demonstrated a link between the GAD-7 scores at baseline and the degree of improvement after three weeks of treatment, suggesting that patients with more severe anxiety may benefit from treatment more than patients with mild-to-moderate anxiety. No adverse events or trends were observed in any patient subgroup.

Nearly a third of all patients were 60 years or older and receiving concomitant therapy. These observations align with prior reports indicating that Adaptol^®^ is well tolerated in older adults and when administered alongside cardiovascular medications, without increasing the risk of adverse events [6]. However, even when Adaptol^®^ was added to cardiovascular therapy regimens, no adverse events were reported. This suggests that the use of Adaptol^®^ in sensitive patient populations (older adults and those with comorbidities and ongoing treatments) is safe and well tolerated within the observed treatment period. Of note, the effect of sex warrants further investigation, as we observed that men exhibit more improvement than women for somatic symptoms and anxiety, however without significance testing. Nevertheless, this difference may indicate either pharmacological factors or psychosocial influences such as help-seeking behaviour, coping styles, or other gender-related factors.

This study has several limitations. First, the data were collected from routine clinical practices with a limited ability to evaluate the potential effects of various confounding factors, including patients’ health and daily habits. The observational, open-label design should be noted, and the findings primarily indicate associations between Adaptol^®^ use and changes in anxiety. Psychological variables, such as concurrent therapy, coping patterns, and other stressors, were not assessed, limiting the interpretation of psychosocial influences on outcomes. In addition, the absence of variables such as education, occupation, or psychological treatment history limits contextual interpretation of study results. Second, there is a gap between the target and observed population, as the study protocol excluded patients with severe anxiety. Third, the findings may be influenced by expectancy effects and self-report bias. Moreover, in the absence of an a priori power calculation, results are presented with effect sizes and 95% confidence intervals and are discussed cautiously. Finally, the long-term outcomes were out of the scope of this study.

## 5. Conclusions

Adaptol^®^ use was associated with improvements in anxiety and somatic symptoms. A positive correlation was observed between improvements in anxiety and higher GAD-7 scores at baseline. Future controlled trials incorporating psychological and behavioural variables are needed to further explore these findings. In particular, the medication’s ability to alleviate severe anxiety should be the subject of further research. Adaptol^®^ was well-tolerated by all study participants. Our findings support the safe and effective use of Adaptol^®^ in managing anxiety and the somatic symptoms of anxiety in diverse patient groups, including sensitive populations.

## Figures and Tables

**Table 1:** Distribution of anxiety severity before and after the three-week treatment course according to GAD-7 scores (numerical data).

Before	After	Total		MCSD * (↓)			MCSD * (↑)	
		n	%	n	%	% Within Group	n	%
Mild	Mild	10	12.7	4	5.1	40.0	0	-
Mild	Moderate	2	2.5	0	-	-	2	2.5
Mild	No	8	10.1	7	8.9	87.5	0	-
Moderate	Mild	37	46.8	34	43	91.9		-
Moderate	Moderate	11	13.9	0	-	-	0	-
Moderate	No	8	10.1	8	10.1	100.0	0	-
Moderate	Severe	3	3.8	0	-	-	0	-
Total	-	79	100	53	67.1	-	2	2.5

* MCSD—minimal clinically significant difference (equals 4 points for GAD-7); ↓, ↑—difference indicating a decrease/increase of GAD-7 score, respectively.

**Table 2:** Patients with severe anxiety at baseline.

Gender	Age	GAD-7 Before Treatment	GAD-7 After Treatment	GAD-7 Change	Somatic Symptoms
Female	25	18	5	−13	Improved significantly
Female	71	18	5	−13	Improved
Female	44	19	6	−13	Improved
Female	35	18	4	−14	Improved significantly
Male	54	19	7	−12	Improved
Female	70	18	4	−14	Improved significantly

## Data Availability

The raw data supporting the conclusions of this article will be made available by the author, without undue reservation.

## Abbreviations

The following abbreviations are used in this manuscript:

ATC	Anatomical Therapeutic Chemical classification
GAD-7	Generalized Anxiety Disorder 7-item scale
MCSD	Minimal clinically significant difference
SPSS	Statistical Package for the Social Sciences

## References

[1] Love A.S., Love R. (2019). Anxiety Disorders in Primary Care Settings. Nurs. Clin. N. Am..

[2] Boland R., Verduin M.L., Ruiz P. (2022). Kaplan & Sadock’s Synopsis of Psychiatry.

[3] American Psychiatric Association (2013). Diagnostic and Statistical Manual of Mental Disorders.

[4] Zāļu Valsts Aģentūra (State Medical Agency). https://dati.zva.gov.lv/zalu-registrs/info/03-0002.

[5] Levin O.S. (2024). Izuchenie effektivnosti i vliyaniya na kachestvo zhizni patsientov s subklinicheskim i klinicheski vyrazhennym trevozhnym rasstroistvom mobil’nogo prilozheniya v sochetanii s terapiei preparatom Adaptol [To study the effectiveness and impact on the quality of life of patients with subclinical and clinically pronounced anxiety disorder of a mobile application in combination with Adaptol therapy]. S.S. Korsakov J. Neurol. Psychiatry.

[6] Kazakov Y.M., Potiazhenko M.M., Nastroga T.V. (2023). Treatment Optimization in Management of Combined Pathology—Arterial Hypertension and Post-COVID Syndrome in Elderly Patients. Wiad. Lek..

[7] World Health Organization (2016). The ICD-10 Classification, 2016 (Latvian Version, 5th ed.).

[8] Spitzer R.L., Kroenke K., Williams J.B.W., Löwe B. Generalized Anxiety Disorder Scale (GAD-7). 2006 (Oriģināls). Local adaptation by Vrubļevska et.al (2022). https://www.rsu.lv/en/psychology-laboratory/test-and-survey-registry.

[9] Toussaint A., Hüsing P., Gumz A., Wingenfeld K., Härter M., Schramm E., Löwe B. (2020). Sensitivity to change and minimal clinically important difference of the 7-item Generalized Anxiety Disorder Questionnaire (GAD-7). J. Affect. Disord..

[10] Bolton D. (2023). A revitalized biopsychosocial model: Core theory, research paradigms, and clinical implications. Psychol. Med..

